# Global Transcriptome Sequencing Using the Illumina Platform and the Development of EST-SSR Markers in Autotetraploid Alfalfa

**DOI:** 10.1371/journal.pone.0083549

**Published:** 2013-12-12

**Authors:** Zhipeng Liu, Tianlong Chen, Lichao Ma, Zhiguang Zhao, Patrick X. Zhao, Zhibiao Nan, Yanrong Wang

**Affiliations:** 1 State Key Laboratory of Grassland Agro-ecosystems, School of Pastoral Agricultural Science and Technology, Lanzhou University, Lanzhou, China; 2 Key Laboratory of Cell Activities and Stress Adaptations, Ministry of Education, School of Life Sciences, Lanzhou University, Lanzhou, China; 3 Plant Biology Division, The Samuel Roberts Noble Foundation, Ardmore, Oklahoma, United States of America; University of New England, Australia

## Abstract

**Background:**

Alfalfa is the most widely cultivated forage legume and one of the most economically valuable crops in the world. The large size and complexity of the alfalfa genome has delayed the development of genomic resources for alfalfa research. Second-generation Illumina transcriptome sequencing is an efficient method for generating a global transcriptome sequence dataset for gene discovery and molecular marker development in alfalfa.

**Methodology/Principal Findings:**

More than 28 million sequencing reads (5.64 Gb of clean nucleotides) were generated by Illumina paired-end sequencing from 15 different alfalfa tissue samples. In total, 40,433 unigenes with an average length of 803 bp were obtained by *de* *novo* assembly. Based on a sequence similarity search of known proteins, a total of 36,684 (90.73%) unigenes were annotated. In addition, 1,649 potential EST-SSRs were identified as potential molecular markers from unigenes with lengths exceeding 1 kb. A total of 100 pairs of PCR primers were randomly selected to validate the assembly quality and develop EST-SSR markers from genomic DNA. Of these primer pairs, 82 were able to amplify sequences in initial screening tests, and 27 primer pairs successfully amplified DNA fragments and detected significant amounts of polymorphism among 10 alfalfa accessions.

**Conclusions/Significance:**

The present study provided global sequence data for autotetraploid alfalfa and demonstrates the Illumina platform is a fast and effective approach to EST-SSR markers development in alfalfa. The use of these transcriptome datasets will serve as a valuable public information platform to accelerate studies of the alfalfa genome.

## Introduction

Alfalfa (*Medicago sativa* L. subsp. *sativa*), a member of the Fabaceae family, is the most widely cultivated forage legume in the world and is the third most valuable crop in the United States, after corn (*Zea mays* L.) and soybean (*Glycine max* (L.) Merr.) [1]. Alfalfa is referred to as “the queen of forage crops” because it is highly productive and stress tolerant and provides a valuable forage crop for livestock. In addition, alfalfa has considerable potential for ethanol production. The stems can be separated from the leaves and used as a biofuel crop, while the leaves can be used as a protein supplement for animals [2]. To improve its rumen digestibility, the value of alfalfa could be enhanced by increasing the cellulose content and decreasing the lignin content in stem cell walls.

Cultivated alfalfa is a perennial, obligate outcrossing, autotetraploid (2*n* = 4*x* = 32) plant with a genome size of 800-900 Mb [3]. Synthetic populations of alfalfa are extremely polymorphic due to their high degree of outcrossing. These features complicate genetic and genomic studies of alfalfa and have led to the development of *M. truncatula* as a model legume. *M. truncatula* is a diploid, self-fertile species with a short life cycle and a small genome (500 Mb) [4]. Several framework genetic linkage maps have been constructed for diploid and tetraploid alfalfa using Restriction Fragment Length Polymorphism (RFLP), Simple Sequence Repeat (SSR), Amplified Fragment Length Polymorphism (AFLP), and Single-dose Restriction Fragments (SDRFs) markers [5,6,7,8,9,10,11,12,13,14], and Quantity Trait Loci (QTLs) associated with agronomic traits within relatively large genomic regions have been identified [11,12,15,16,17,18,19,20,21,22,23]. Several studies attempted to transfer EST-SSRs from *M. truncatula* to *M. sativa*. However, only a small number of the EST-SSR markers were polymorphic and could be incorporated into the autotetraploid map of *M. sativa* [11,23].

RNA-Seq, a massively parallel sequencing method for transcriptome analysis, only analyzes transcribed portions of the genome, avoiding the non-coding and repetitive sequences that comprise much of the genome. Recently, RNA-Seq has expanded the identification of alfalfa genes. Using Illumina sequencing, 124,025 unique sequences from MSGI 1.0 have been identified from the elongating stem and post-elongation stem internodes of two alfalfa genotypes with divergent cellulose and lignin concentrations [24]. Using 454 sequencing, 54,216 unique sequences were obtained from the roots and shoots of two alfalfa genotypes with different water stress sensitivities [25]. Illumina sequencing of old and young stems of 27 alfalfa genotypes led to the identification of 25,183 contigs [26]. Additionally, Illumina sequencing was performed in alfalfa roots contrasting in salt tolerance, and 60,290 tentative consensus sequences were obtained [27]. While these experiments have identified numerous transcripts, the transcripts were derived from limited tissues, such as stems, roots, and shoots. These transcriptomes do not encompass the complete set of transcripts in other organs or tissue systems during the different developmental periods of alfalfa. Furthermore, the identification of leaky transcripts may be essential to elucidate the functional elements of alfalfa and reveal the molecular characteristic of cells and tissues. Therefore, sequencing the transcriptome of a broader array of tissues will permit the global identification of transcripts that may be useful in modern alfalfa breeding programs.

In the present study, we performed *de novo* transcriptome sequencing of M. sativa L. subsp. *sativa* using the Illumina GA IIx sequencing platform. A total of 15 tissue types were included to identify the global and complete transcriptome of this species. The transcriptome coverage was 5.64 Gb of clean nucleotides, which was sufficiently comprehensive to identify most known genes and major metabolic pathways. A total of 40,433 unigenes were identified, and 1,649 SSRs were determined. This dataset is a public information platform for gene expression analysis, genomics, and functional genomics in M. sativa L. subsp. *sativa*. 

## Materials and Methods

### Tissue Material and RNA Isolation

**Figure 1:**
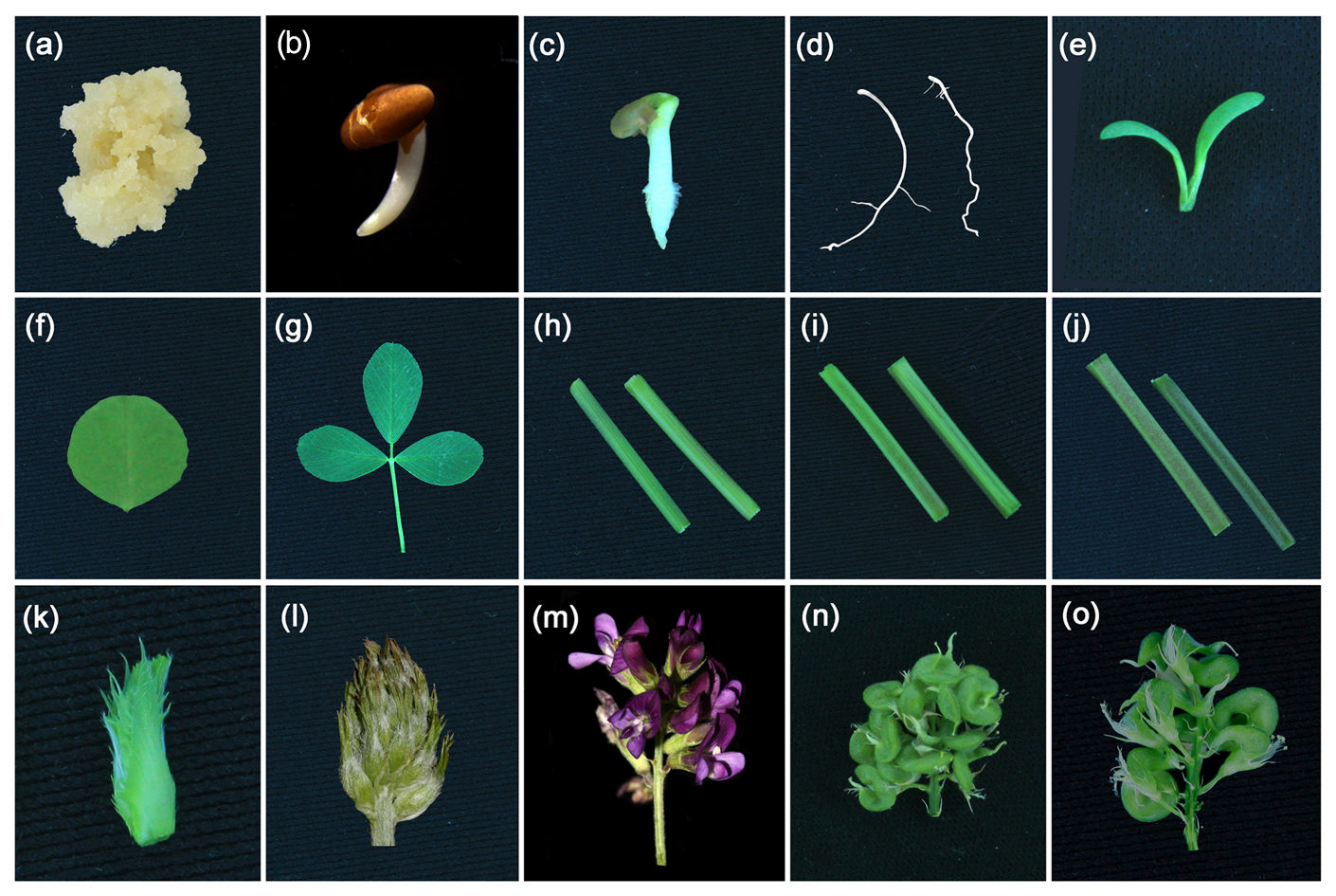
The tissues used in this study and all samples shown are from the alfalfa cultivar “Golden queen”. (A) Callus cells. (B) Germinated seeds (36 hours after seed germination). (C) Germinated seeds (48 hours after seed germination). (D) Roots from a 20-day-old seedling. (E) Cotyledons from a 7-day-old seedling. (F) Unifoliate leaves from a 20-day-old seedling. (G) Compound leaves. (H) Young stem (less lignified). (I) Middle stem (moderately lignified). (J) Old stem (more lignified). (K) Shoot apex. (L) Young inflorescence (diameter 0.4-0.5 cm). (M) Mature inflorescence (diameter 2 cm), (N) Young pod (16 days after pollination). (O) Mature pod (24 days after pollination).

The alfalfa cultivar “Golden queen” was grown in a greenhouse under a 16 h light/8 h dark cycle at 22°C at Lanzhou University, Lanzhou, China. A total of 15 tissue types were collected, including germinated seeds (36 hours after seed germination), germinated seeds (48 hours after seed germination), cotyledons (from a 7-day-old seedling), unifoliate leaves (from a 20-day-old seedling), roots (from a 20-day-old seedling), compound leaves, young stems (less lignified), middle stems (moderately lignified), old stems (highly lignified), shoot apex, young inflorescences (diameter 0.4-0.5 cm), mature inflorescences (diameter 2 cm), young pods (16 days after pollination), and mature pods (24 days after pollination) (Figure 1). Callus cells were induced from alfalfa seeds on MS solid medium containing 2,4-D (2.0 mg/L) and 6-BA (0.5 mg/L) under a 16 h light/8 h dark cycle at 25°C for approximately 30 days. The callus cells were actively dividing and consisted of a mass of undifferentiated cells. All sampled tissues were immediately frozen in liquid nitrogen and stored at -80°C until RNA extraction.

For Illumina sequencing, the total RNA from each sample was isolated using the RNeasy Plant Mini Kit (Qiagen, Cat. # 74904). Both the quantity and quality of the RNA were verified using NanoDrop ND1000 (Thermo Scientific) and Agilent 2100 Bioanalyzer (Agilent). The total RNA from all 15 samples was adjusted to the same concentration. To cover more tissue-specific transcripts in alfalfa, a total of 20 µg of RNA was pooled equally from the 15 tissues for cDNA library preparation.

### cDNA Library Construction and Sequencing

The cDNA library was constructed following the manufacturer’s instructions for the mRNA-Seq Sample Preparation Kit (Cat # RS-930-1001, Illumina Inc., San Diego, CA). Briefly, the total RNA was collected and pooled from all 15 alfalfa tissues, and magnetic oligo(dT) beads were used to isolate the poly(A) mRNA. An RNA Fragmentation Kit (Ambion, Austin, TX) was used to cleave the mRNA into short fragments, which were then used as templates to reverse-transcribe first-strand cDNA using random hexamer primers and reverse transcriptase (Invitrogen, Carlsbad, CA). Second-strand cDNA was synthesized in a reaction containing buffer, dNTPs, RNase H (Invitrogen) and DNA polymerase I (New England BioLabs, Ipswich, MA). A paired-end library was synthesized using the Genomic Sample Preparation Kit (Illumina) according to the manufacturer’s instructions. Short fragments were purified with the MinElute PCR Purification Kit (QIAGEN, Dusseldorf, Germany) and eluted in 10 µL of EB buffer (QIAGEN). The short fragments were connected with sequencing adapters, and the desired fragments (200 ± 25 bp) were separated by agarose gel electrophoresis and purified with a gel extraction kit. Finally, the sequencing library was constructed by PCR amplification (15 cycles) and sequenced using the Illumina Genome Analyzer IIx sequencing platform. Data analyses and base calling were performed with the Illumina instrument software. The transcriptome datasets are available in the NCBI Sequence Read Archive (SRA) under accession number SRX247927.

### Sequence Analyses, Assembly, and Annotation

All raw reads were cleaned by removing adapter sequences, low-quality sequences with ambiguous bases ‘N’, and reads with more than 10% Q < 20 bases. *De novo* transcriptome assembly of these quality reads was performed using the Trinity software, which recovers more full-length transcripts across a broad range of expression levels with a sensitivity similar to methods that rely on genome alignments [28]. The overlap settings used for the Trinity assembly were 31 bp with 80% similarity, and all other parameters were set to default values.

For further analysis, we first used BLASTX (E-value <10E-5) to compare the assembled sequences against the NCBI Nr, Nt, and Swiss-Prot databases. Gene names were assigned to each assembled sequence based on the highest scoring BLAST hit. To annotate the assembled sequences with Gene Ontology (GO) terms describing biological processes, molecular functions and cellular components, we used the BLAST2GO program to obtain the GO annotations of the unigenes [29]. The annotated unigenes were enriched and refined using ANNEX [30]. 

The unigene sequences were also aligned to the Clusters of Orthologous Group (COG) database. The Kyoto Encyclopedia of Genes and Genomes (KEGG) pathways were assigned to the assembled sequences using online software (http://www.genome.jp/kegg/kegg4.html) to predict and classify functions. We used the bi-directional best hit (BBH) method to obtain KEGG Orthology (KO) assignments [31]. 

We also performed a homologous comparison of the genomes of alfalfa and *M. truncatula*. The genome sequence data for *M. truncatula* were obtained from the Mt3.5.2 release (http://tofu.cfans.umn.edu/). Based on nucleotide sequence similarity, a BLAST search was performed with a cutoff E-value <10E-10 using blast-2.2.23-ia32-win32.

### Detection of EST-SSR Markers and Primer Design

SSRs were detected in the 40,433 alfalfa unigenes with the Simple Sequence Repeat Identification Tool program (SSRIT, http://www.gramene.org/db/markers/ssrtool). Only unigenes that were longer than 1 kb were included in the EST-SSR detection. The parameters were adjusted to identify perfect di-, tri-, tetra-, penta-, and hexa-nucleotide motifs with a minimum of 6, 5, 5, 5, and 5 repeats, respectively. The EST-SSR primers were designed using BatchPrimer3 (http://probes.pw.usda.gov/cgi-bin/batchprimer3/batchprimer3.cgi).

### EST-SSRs Amplification and Diversity Analysis

A total of 10 alfalfa accessions including Chinese landraces, cultivars, and foreign collections (Table S1) were selected for polymorphism investigation with the EST-SSRs. Total genomic DNA was extracted from young leaves of five individual plants in each accession via the cetyltrimethylammonium bromide (CTAB) method [32]. PCR amplifications were conducted in a final volume of 10 µL containing 40 ng template DNA, 1× PCR buffer, 2.0 mM MgCl_2_, 2.5 mM dNTPs, 4 µM each primer, and 0.8 U Taq polymerase (TaKaRa). The PCR reaction cycling included 4 min at 94°C, 35 cycles of 30 s at 94°C, 35 s at the annealing temperature (Table S2), and 1 min at 72°C, with a final extension step of 5 min at 72°C. The PCR products were subjected to electrophoresis on 8.0% non-denaturing polyacrylamide gels and stained by ethidium bromide [33]. The band sizes were determined by comparison with the DL500 DNA maker (TaKaRa). The observed heterozygosity (Ho) was calculated as previous described [34,35], and the corrected heterozygosity (*He*, corrected for sample size) and Shannon-Wiener diversity index (*H*’*c*, corrected for sample size) were analyzed by the ATETRA 1.2.a software program [36] . Only specific bands that could be unambiguously scored across all individual plants were used in this study. The resulting data matrix was analyzed using POPGENE v. 1.31 [37].

## Results

### Illumina Sequencing and *de novo* Assembly

To globally and comprehensively cover the alfalfa transcriptome, total RNA was extracted from 15 different alfalfa tissues. The morphology of each tissue is shown in Figure 1. Equal amounts of total RNA from each alfalfa tissue were pooled together. The cDNA was subjected to Illumina Genome Analyzer sequencing. After stringent quality assessment and data filtering, 28,790,610 reads (5.64 Gb) with 94.48% Q20 bases were selected as high-quality reads for further analysis. An overview of the sequencing results is presented in Table 1. The high-quality reads were deposited into the NCBI SRA database (SRX247927).

**Table 1:** Summary of Illumina transcriptome sequencing for alfalfa.

**Sample**	**Read length**	**No. of Reads**	**Data**	**GC (%)**	**Q20 (%)**
Alfalfa	90	28,790,610	5,642,959,560	47.61	99.48

**Figure 2:**
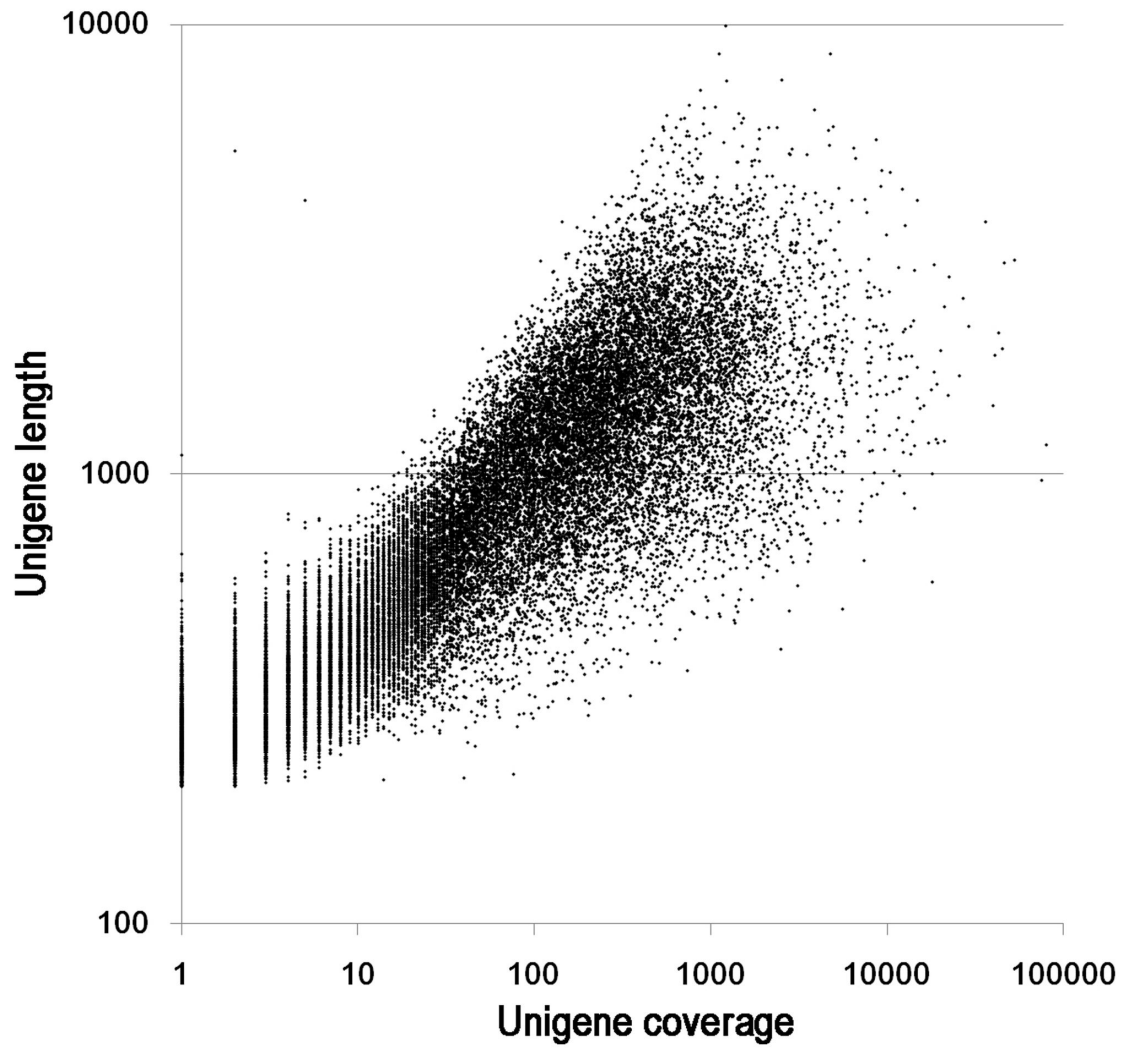
Plot showing the dependence of unigene length on the number of reads assembled into each unigene.

All high-quality reads were assembled *de novo* using the Trinity program [28], which produced 81,277 scaffolds with an N50 length of 1,323 bp and a mean length of 873 bp. The distribution of the scaffolds is shown in Table 2. A total of 40,433 unigenes were obtained with an N50 length of 1,300 bp and a mean length of 803 bp. The length distributions of the unigenes are shown in Table 2. As expected for a randomly fragmented transcriptome, there was a positive relationship between the length of a given unigene and the number of reads assembled into it (Figure 2).

**Table 2:** Length distribution of scaffolds and unigenes.

**Nucleotide length (bp)**	**scaffolds**	**unigenes**
0-100	0(0)	0(0)
100-200	0(0)	0(0)
200-300	17,764(21.86)	11,280(27.9)
300-400	10,692(13.16)	5,922(14.65)
400-500	7,449(9.16)	3,688(9.12)
500-600	5,554(6.83)	2,420(5.99)
600-700	4,653(5.72)	1,888(4.67)
700-800	4,019(4.94)	1,629(4.03)
800-900	3,462(4.26)	1,446(3.58)
900-1,000	3,072(3.78)	1,259(3.11)
1,000-1,200	5,234(6.44)	2,256(5.58)
1,200-1,400	4,268(5.25)	1,827(4.52)
1,400-1,600	3,403(4.19)	1,478(3.66)
1,600-1,800	2,725(3.35)	1,280(3.17)
1,800-2,000	2,090(2.57)	951(2.35)
2,000-2,200	1,594(1.96)	749(1.85)
2,200-2,400	1,152(1.42)	518(1.28)
2,400-2,600	903(1.11)	405(1)
2,600-2,800	693(0.85)	326(0.81)
2,800-3,000	539(0.66)	237(0.59)
>3,000	2,011(2.47)	874(2.16)
Total	81,277	40,433
Minimum length (bp)	201	202
Maximum length (bp)	15,236	15,237
N50 (bp)	1,323	1,300
Average length (bp)	873	803
Total nucleotide length (bp)	70,966,536	32,482,946

**Figure 3:**
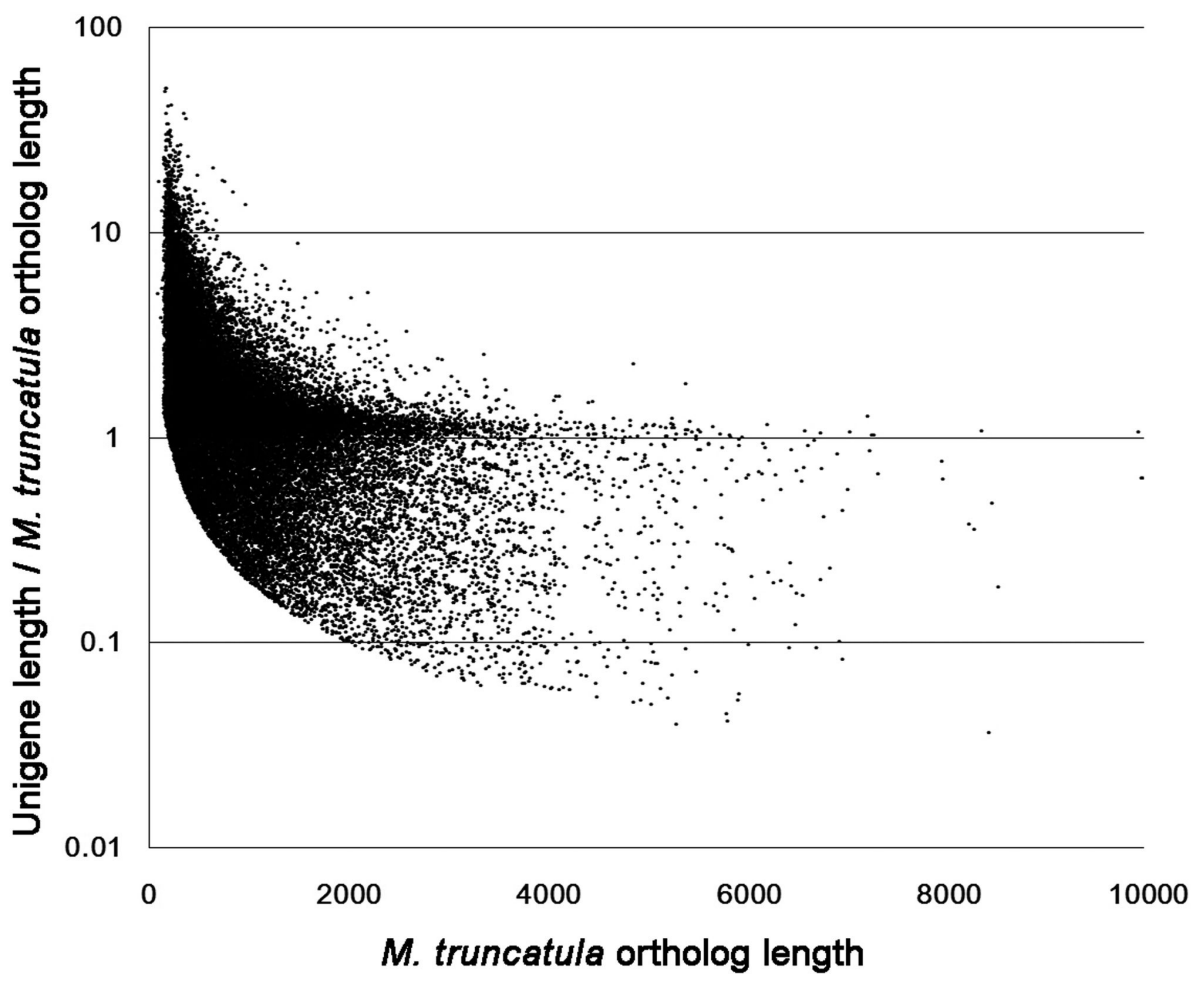
The ratio of *M. sativa* unigene length to *M. truncatula* unigene coverage depth. A BLAST search was performed with a cutoff E-value <10E-10 between *M. truncatula* and *M. sativa* on the basis of coding sequence similarity.

To further assess the extent of transcript coverage provided by the unigenes, we plotted the ratio of assembled unigene length against *M. truncatula* ortholog length (Figure 3). Based on the similarity of coding sequences, a BLAST search was performed with a cutoff E-value <10E-10 between *M. truncatula* and *M. sativa*. Among the 64,127 (Mt3.5.2) transcripts, 41,447 (64.63%) *M. truncatula* transcripts had homologous transcripts in the *M. sativa* genome. This finding suggests that most of the *M. truncatula* ortholog coding sequences were covered by at least one individual unigene. The plotting results revealed that 14,933 *M. truncatula* orthologs (36.03%) were covered by unigenes with a percentage less than 100%, and 26,514 orthologs (63.93%) were covered by unigenes with a percentage greater than 100% (Figure 3). This finding indicates that additional sequencing and more advanced assembly technology are needed for more comprehensive and precise transcriptome data in alfalfa.

**Figure 4:**
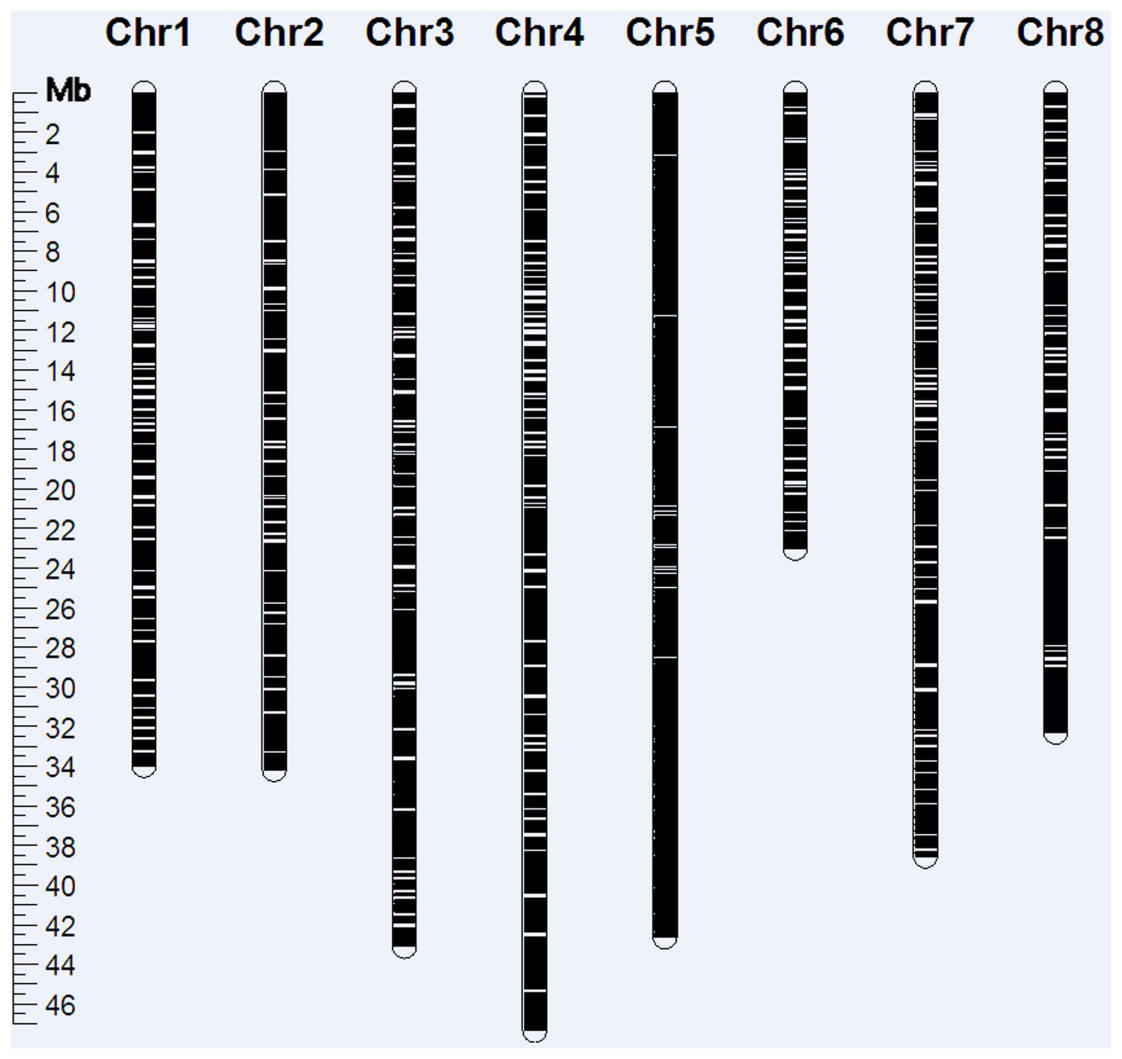
Alfalfa unigenes mapped to Mt3.5.2 pseudomolecules with a threshold of 95% identity and 90% coverage. White lines represent alfalfa unigenes that were not mapped to the *M. truncatula* genome sequence assembly, and black lines represent alfalfa unigenes that were mapped to the *M. truncatula* genome.

In addition, the available *M. truncatula* genome sequence was used as a scaffold to align the alfalfa unigene sequences. Under stringent conditions using Blat, including a threshold of 95% identity and 90% coverage (Figure 4), 27,853 (68.89%) unigenes were mapped to the Mt3.5.2 genome sequence assembly, and their likely map positions were inferred. Among the nine chromosomes in *M. truncatula*, chromosome 5 (4,348) and 6 (1,491) showed the highest and the lowest number of unigenes on the Mt3.5.2 chromosomes, respectively (Table 3).

**Table 3:** Summary of the unigenes and their location on the Mt3.5.2 chromosomes.

Chr1	Chr2	Chr3	Chr4	Chr5	Chr6	Chr7	Chr8	Chr0	Total
3,128	3,069	3,841	4,047	4,348	1,491	3,363	2,969	1,597	27,853

### Functional Annotation

Unigene annotations provide functional information, including protein sequence similarities, GO terms, COG clusters, and KEGG pathway information. The unigenes were annotated by alignment with the following databases: the National Center for Biotechnology Information (NCBI) nonredundant protein (Nr) database, the NCBI non-redundant nucleotide sequence (Nt) database, Swiss-Prot, KEGG, COG, the InterPro (Ipr) database, and TrEMBL. The best alignment was selected from the matches with E-values less than 10E-5. The overall functional annotations are depicted in Table 4. Altogether, 36,684 (90.73%) unigenes were successfully annotated in the Nr, Nt, Swiss-Prot, KEGG, COG, Ipr, and TrEMBL databases, suggesting that they have relatively well-conserved functions (Table 4). According to the RPKM, 5,000 unigenes were chosen as highly expressed unigenes, of which 4,941 unigenes were annotated by the NCBI Nr protein database (Table S3).

**Table 4:** Functional annotation of the alfalfa transcriptome.

**Annotated database**	**Annotated number**	**300-1000 bp**	**≥1000 bp**
Nr Annotation	29,026	13,332	10,746
Nt Annotation	34,695	15,681	10,790
Swissprot Annotation	21,122	8,913	9,348
GO Annotation	14,415	5,511	7,490
KEGG Annotation	5,723	2,163	2,825
COG Annotation	8,522	2,984	4,838
Ipr Annotation	19,166	7,868	9,293
TrEMBL Annotation	31,258	14,443	10,806
Total	36,684	16,782	10,885

**Figure 5:**
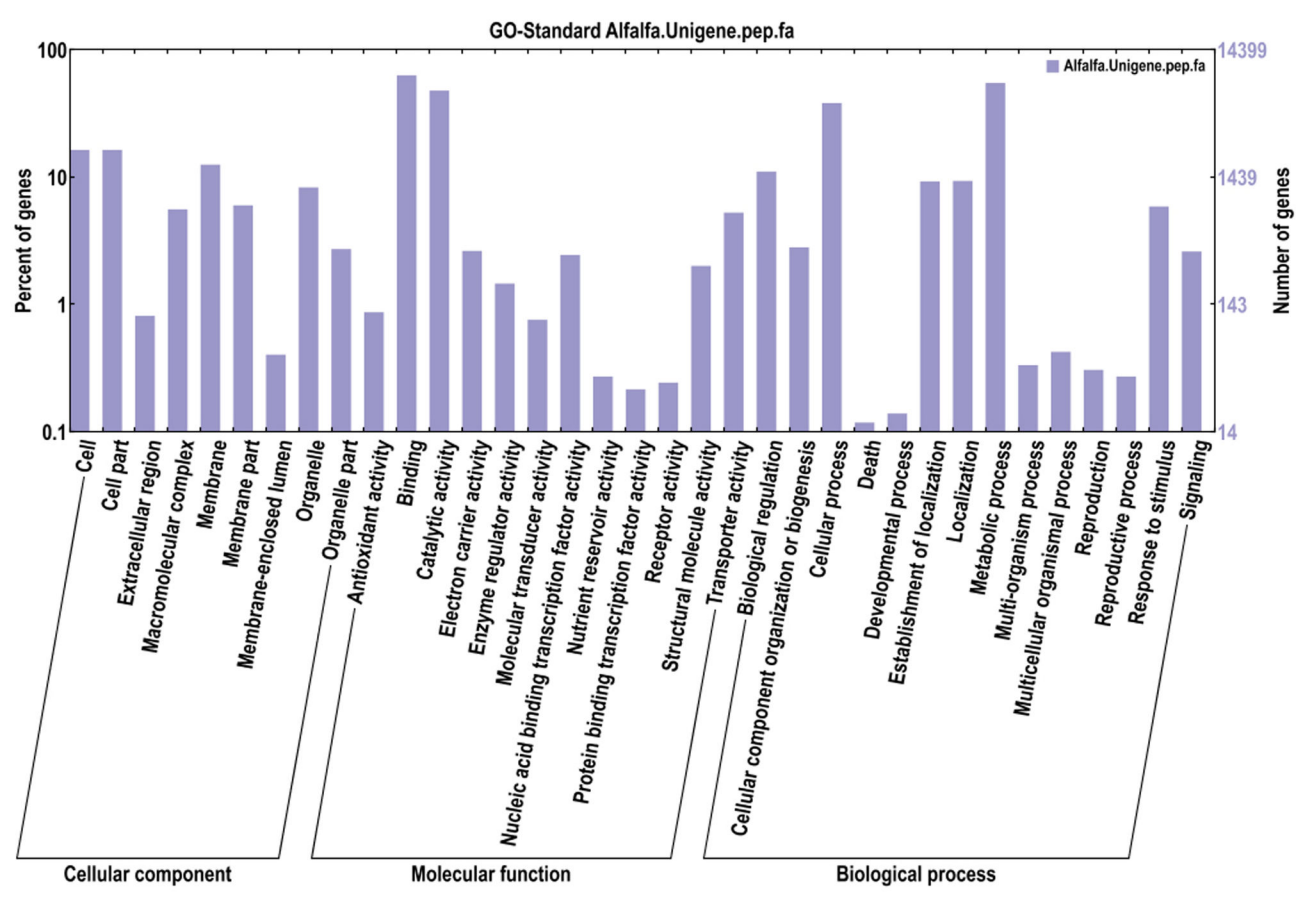
Gene Ontology (GO) classification of assembled unigenes in *M. sativa*. A total of 14,415 unigenes with BLAST matches to known proteins were assigned to three main categories: cellular component, molecular function and biological process.

Based on 29,026 Nr annotations, 14,415 (35.65%) unigenes were assigned to one or more GO terms. The GO-annotated unigenes belonged to the biological process, cellular component, and molecular function clusters and were distributed across 35 categories, with 34.4% in biological processes, 50.7% in molecular functions, and 14.9% in cellular components (Figure 5). In the biological processes category, metabolic processes (GO:0008152) was the dominant group, followed by cellular processes (GO:0009987), biological regulation (GO:0065007). With regard to molecular functions, the majority of unigenes were assigned to binding (GO:0005488), followed by catalytic activity (GO:0003824) and transporter activity (GO:0005215). For the cellular components category, cell (GO:0005623) and cell part (GO:0044464) were highly represented (Figure 5).

**Figure 6:**
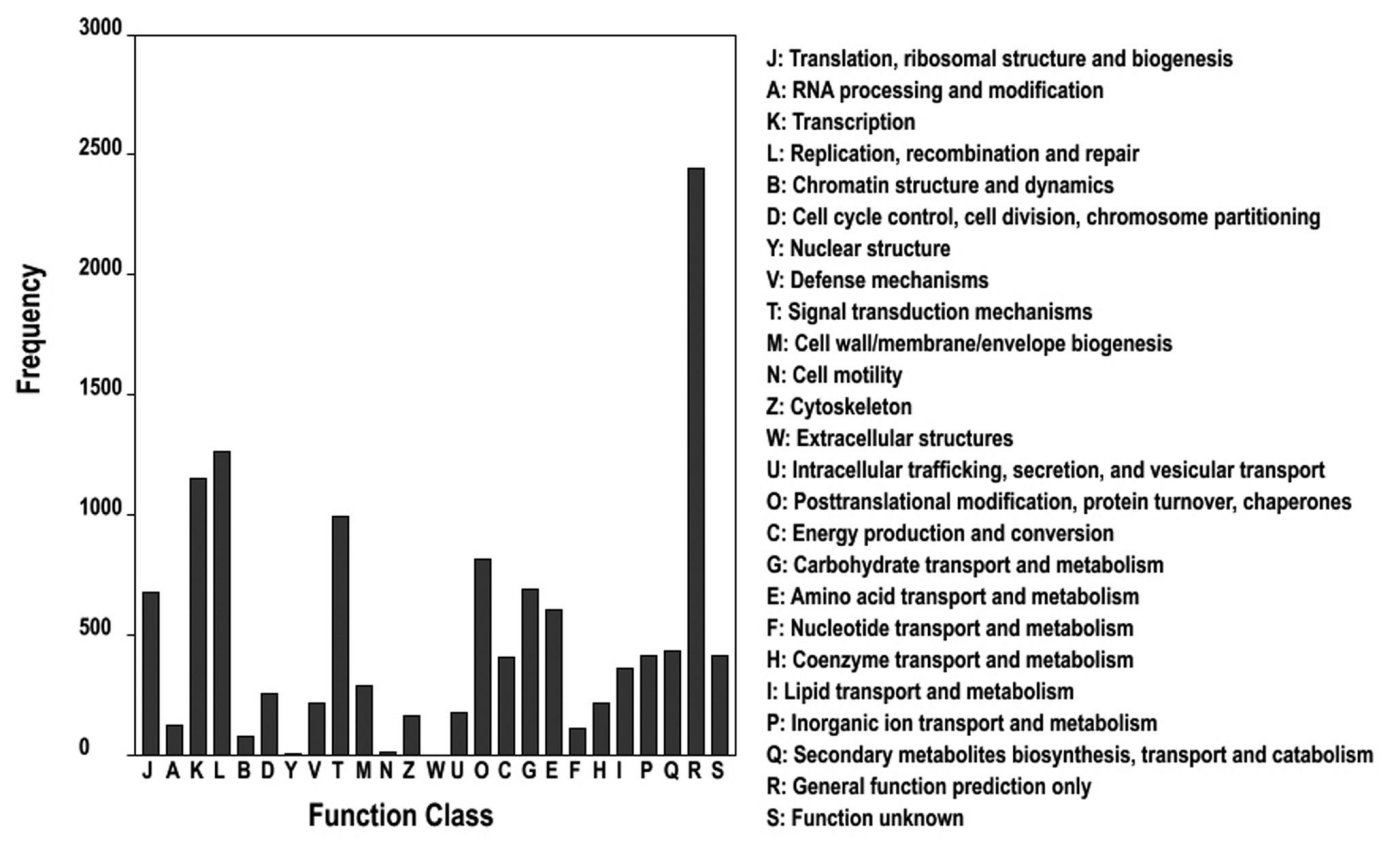
Clusters of orthologous groups (COG) classification. In total, 8,522 of the 40,433 sequences with Nr hits were grouped into 25 COG classifications.

In addition, all 40,433 unigenes were subjected to a search against the COG database. Of 29,026 unigenes with significant similarity to nr proteins in this study, 8,522 (21.08%) were assigned to COG classifications (Figure 6). Among the 25 COG categories, the cluster for general function prediction represented the largest group, followed by replication, recombination and repair, etc. (Figure 6). To further analyze the alfalfa transcriptome, all 40,433 unigenes were analyzed with KEGG pathway tools. This process predicted a total of 293 pathways that were represented by 5,723 (14.15%) unigenes (Table S4).

### SSR Discovery and Polymorphic Analysis

To further assess the assembly quality and to develop new molecular markers, all 40,433 alfalfa unigenes that were identified in the present study were used to mine potential microsatellites, which are defined as di- to hexa-nucleotide SSRs with a minimum of five repeats for all motifs. First, 10,901 unigenes with lengths greater than 1 kb were screened. Using the SSRIT tool, a total of 1,649 potential EST-SSRs were identified from 1,494 unigenes, of which 185 sequences contained more than one EST-SSR (Table 5). To identify the putative functions of the genes containing the SSR loci, 1,494 unigenes were searched against the Nt database with an E-value less than 10E-5. Of these queries, 1,485 unigenes had BLAST hits to known proteins in this database (Table S5).

**Table 5:** Summary of EST-SSR types in the alfalfa transcriptome.

**Repeat motif**	**Number** ^a^	**Percentage** ^b^
Di-nucleotide		
AC/AG/AT	136	
CA/CT/CG	81	
TA/TC/TG	74	
GA/GC/GT	107	
Total	398	24.14
Tri-nucleotide		
AAC/AAT/AAG/ACA/ACC/ACG/ACT	118	
ACA/ACC/ACG/ACT/ATA/ATC/ATG/ATT	152	
CAA/CAC/CAG/CAT/CCA/CCT/CCG	130	
CGA/CGC/CGG/CGT/CTA/CTC/CTG/CTT	61	
GAA/GAC/GAG/GAT/GCA/GCC/GCG/GCT	143	
GGA/GGC/GGT/GTA/GTC/GTG/GTT	83	
TAA/TAC/TAG/TAT/TCA/TCC/TCG/TCT	135	
TGA/TGC/TGG/TGT/TTA/TTC/TTG	187	
Total	1,009	61.19
Tetra-nucleotide		
AAAC/AACA/AAGA/ACAG/ACAT/ACTC	7	
ATAG/ATCT/ATGA/ATGT/ATTT/CAAC	7	
CATG/CGTG/CTAT/GAAA/GAAC/GAAT	6	
GACG/GATA/GATT/GGTT/TAGA/TATC	6	
TATT/TCAT/TCGT/TCTG/TCTT/TGAA	6	
TGAG/TTAA/TTCA/TTCT/TTGT/TTTA/TTTC	9	
Total	41	2.49
Penta-nucleotide		
AAAGA/AAGAA/CCTGC/CGTTT	4	
GAAAT/TCTGT/TGAGA/TTCTC	4	
TTGTA/TTTCA/TTTTG/	3	
Total	11	0.67
Hexa-nucleotide		
GGATTT/ATCCTC/ATGAAC	3	
AGCAGG/CTCTTC/	2	
Total	5	0.30
Compound SSRs	185	11.22
Sum	1,649	

^a^ Number of total SSRs detected in unigenes, ^b^ percentage of total SSRs with different repeat motifs.

The frequency, type and distribution of the 1,649 potential SSRs were analyzed. We identified SSRs only from unigenes with lengths greater than 1 kb. For these unigenes, an average of one SSR was found every 12.06 kb, and the frequency of SSRs was 15.13%. As shown in Table 5, tri-nucleotide repeats were the most abundant type (1,009; 61.19%), followed by bi- (398; 24.14%), tetra- (41; 2.49%), penta- (11; 0.67%), and hexa-nucleotide (5; 0.30%) repeats. Di- to hexa-nucleotide motifs were analyzed for the number of repeat units. As shown in Table 6, we found that the most highly represented repeat unit of potential SSRs was 5, which accounted for 689 (47.06%), followed by 6 (373; 25.48%), 7 (161; 11.41%), and 8 (41; 2.80%). The SSR length was mostly distributed from 12 to 20 bp, accounting for 86.54% of the total SSRs. In total, 122 motif sequence types were identified, among which, di-, tri-, tetra-, penta-, and hexa-nucleotide repeats had 11, 58, 37, 11, and 5 types, respectively. The AG/CT repeat was the most abundant motif detected in all SSRs (188; 12.84%), followed by GAA/TTC (143; 9.77%).

**Table 6:** Length distribution of EST-SSRs based on the number of repeat units.

**Number of repeat units**	**Di-**	**Tri-**	**Tetra-**	**Penta-**	**Hexa-**	**Total**	**Percentage**
5	0	646	31	8	4	689	47.06
6	118	244	8	2	1	373	25.48
7	59	107	1	0	0	167	11.41
8	60	10	1	0	0	71	4.85
9	41	0	0	0	0	41	2.8
10	68	0	0	1	0	69	4.71
≥10	52	2	0	0	0	54	3.69

Based on the SSR-containing sequences, 100 pairs of EST-SSR primers were designed using BatchPrimer3. Detailed information about the designed primers is shown in Table S2. Of the 100 primer pairs, 82 were able to amplify PCR products from alfalfa genomic DNA, while 18 primer pairs failed to amplify PCR products at various annealing temperatures and Mg^2+^ concentrations. Of the 82 successful primer pairs, 37 PCR products were of the expected sizes, and 34 primer pairs generated PCR products that were larger than expected. The PCR products of the other 11 primer pairs were smaller than expected. Of the 37 primer pairs that amplified PCR products of the expected sizes, 27 PCR amplifications resulted in more than one band, which might be due to the high heterozygosity of the autotetraploid alfalfa germplasm.

**Figure 7:**
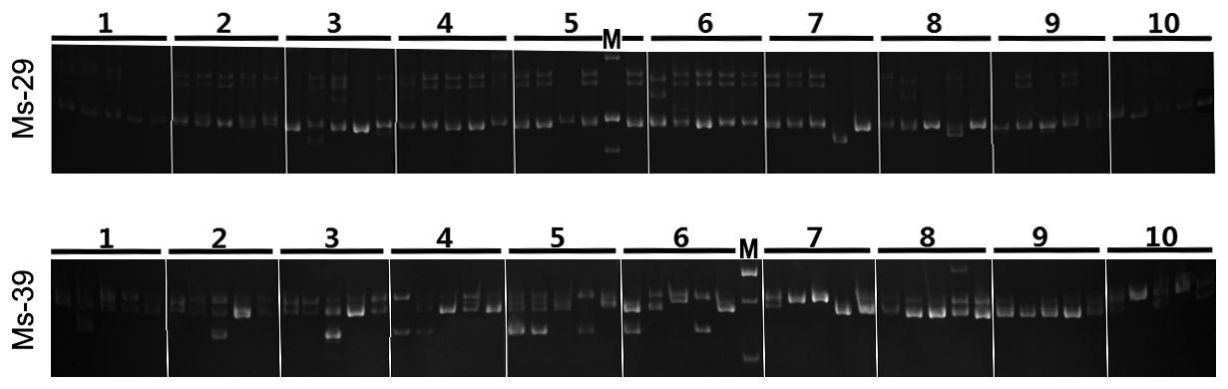
EST-SSR variations at the Ms-29 and Ms-39 loci of 10 alfalfa accessions. Each accession includes five individual plants. The corresponding detailed information from 10 alfalfa accessions is displayed in Table S1. The letter ‘M’ denotes the molecular markers, which are 300 bp, 200 bp, and 150 bp (top to bottom) in Ms-29, and 200 bp, 150 bp, and 100 bp in Ms-39.

These 27 primer pairs were used to evaluate polymorphisms across 10 alfalfa accessions (Table 7 and Figure 7), with five individual plants in each accession. All 27 EST-SSR loci showed different allelic polymorphisms. The number of alleles per locus varied from three to 11 (mean: 6.11). The mean values of Ho, He, and *H*’*c* was 0.64, 0.64, and 1.23 (Table 7). The maximum genetic distance value was 0.1517 between the Longdong I and AmeriStand 403T accessions and the minimum value of 0.0271 between the Longdong III and Xinjiangdaye accessions (Table S6).

**Table 7:** Characterization of 27 EST-SSRs in 10 alfalfa accessions.

**Primer**	**Tm (°C)**	**Size range (bp)**	**No. of alleles**	***Ho***	***He***	***H’c***
Ms-02	51	153-171	3	0.61	0.54	0.84
Ms-03	54	202-211	3	0.09	0.17	1.49
Ms-06	57	191-245	6	0.91	0.79	1.38
Ms-07	53	206-308	6	0.42	0.60	1.59
Ms-10	52	192-200	3	0.57	0.54	1.46
Ms-15	51	175-271	4	0.52	0.61	1.72
Ms-21	56	157-265	9	0.93	0.78	1.36
Ms-22	54	156-204	6	0.90	0.78	1.42
Ms-23	54	219-240	6	0.26	0.84	1.63
Ms-27	51	170-257	7	0.88	0.82	1.20
Ms-29	54	160-274	11	0.68	0.81	1.00
Ms-30	54	227-350	6	0.64	0.74	0.40
Ms-34	54	184-252	9	1.00	0.79	0.84
Ms-39	55	126-166	8	0.84	0.72	1.38
Ms-40	53	168-237	8	0.89	0.78	0.66
Ms-41	52	166-180	3	1.00	0.37	1.30
Ms-43	55	162-219	9	0.91	0.78	0.99
Ms-47	54	159-231	6	0.25	0.57	0.61
Ms-50	55	130-259	8	0.76	0.73	1.33
Ms-51	52	147-215	5	0.63	0.53	1.77
Ms-64	51	158-260	5	0.55	0.51	1.20
Ms-66	51	184-283	7	0.48	0.67	1.50
Ms-67	52	185-275	7	0.66	0.67	1.10
Ms-68	54	147-273	7	0.66	0.76	1.32
Ms-74	53	205-235	4	0.17	0.27	1.21
Ms-76	54	178-212	5	0.74	0.61	1.52
Ms-96	56	181-268	4	0.42	0.56	1.52
Mean			6.11	0.64	0.64	1.23

The corresponding detailed information for the 27 primers is displayed in Table S2.

## Discussion

### Illumina Paired-end Sequencing

Transcriptome sequencing is one of the most effective tools for identifying transcript sequences, which is essential for identifying novel genes and developing molecular markers. Next-generation sequencing (NGS) technologies have provided opportunities for high-throughput gene discovery on a genome-wide scale with or without a complete genome sequence. Because of its high efficiency, speed, accuracy, and low cost, NGS has been widely used to analyze transcriptome sequencing and assembly in many non-model plants such as pine [38], ginseng [39], *Artemisia annua* [40], common vetch [41], bamboo[42], sweet potato [43], sesame [35], rubber [44], safflower [45], and alfalfa [24].

Prior to the present study, all alfalfa sequencing efforts using RNA-Seq and 454 sequencing were based on a limited number of tissues, such as stems, roots, and shoots [24,25,26]. In the previous studies, some homologous transcripts specifically expressed in other tissues might be leaky. Here, we used the Illumina HiSeqTM 2000 platform to profile the alfalfa transcriptome from 15 different tissues, obtaining 5.64 Gb clean reads and identifying 40,433 unigenes from a *de novo* assembly. Of the 40,433 unigenes, 36,684 (90.73%) were successfully annotated, indicating their conserved functions. This procedure produced unigenes with an average length of 803 bp; the alfalfa unigenes produced in previous studies had average lengths of 541 bp [25] and 384 bp for unique sequences [24] in alfalfa. 

In the present study, homologs of 71.8% (29,026 of 40,433) of the alfalfa unigenes were identified by searching with BLASTX against the Nr database, whereas in sweet potato [43], bamboo [42], sesame [35], and safflower [45], only 48.54%, 67.5%, 53.91%, and 65.3% of unigenes, respectively, had homologs in the Nr database. 

Altogether, 90.73% of the unigenes were annotated in the Nr, Nt, Swiss-Prot, KEGG, COG, Ipr, and TrEMBL databases. The low percentage (9.3%; 3,749 of 40,433) of unmapped unigenes that were assigned a putative function might be mainly due to the relatively short sequences of the resulting unigenes, most of which likely lack conserved functional domains [46]. Alternatively, these sequences might contain a known protein domain but not display sequence matches due to the short query sequence, resulting in false-negative results. Specifically, 20.1% of unigenes shorter than 300 bp, 8.1% of unigenes between 300 bp and 1,000 bp and 0.1% of unigenes longer than 1,000 bp had no BLAST matches. Another potential reason for a lack of matches is that some of these short unigenes represent non-coding RNAs. The insufficient number of ESTs and limited genomic information for alfalfa in public databases also influences the annotation efficiency. Furthermore, a minority of unigenes without BLAST hits might function as autotetraploid alfalfa-specific genes.

The alfalfa unigenes were assigned to a wide range of GO categories and COG classifications, indicating that our paired-end sequencing data represented a wide diversity of transcripts (Figures 5 and 6). Most representative unigenes were mapped by the KEGG database to specific pathways, such as plant hormone signal transduction, amino acid metabolism, lipid metabolism, energy metabolism, plant-pathogen interactions, photosynthesis, and transcription factors (Table S4). Among the GO categories, cell, binding activity, and metabolic process were the most abundant classes in the cellular component, molecular function, and biological process categories, respectively; these results are consistent with studies in sweet potato [43], rubber [44], and sesame [35]. 

We generated a large transcriptome that can be used for novel gene discovery or for the investigation of alfalfa molecular mechanisms. Our results confirm that high-throughput Illumina paired-end sequencing is an efficient, inexpensive, and reliable method for transcriptome analysis in non-model plants, including autotetraploid plants.

### SSR Markers Identification and Characterization

SSRs are powerful molecular markers that are locus-specific, co-dominant, multi-allelic, highly polymorphic, and transferable among species within genera [47]. EST-SSR markers are very important for studies involving genetic diversity, cultivar identification, evolution, linkage mapping, QTL mapping, comparative genomics, and marker-assisted selection breeding [26]. Most alfalfa SSR markers are derived from *M. truncatula*, with the exception of 61 polymorphic genomic SSRs [48] and 29 polymorphic EST-SSRs [49] developed from alfalfa. Therefore, the development of novel SSR markers from alfalfa could be more useful for genetic studies and breeding applications. The transcriptome sequencing provided many of sequences for developing numerous EST-SSRs. Although we used stringent selection criteria 1,649 potential EST-SSRs were identified in 1,494 unigenes. Tri-nucleotide repeats were the most abundant motif type, followed by di-, tetra-, penta-, and hexa-nucleotide repeats, which is consistent with previous reports [49]. 

Of 100 PCR primer pairs that were randomly selected for validation, 82 amplified clear bands. Our PCR success rate was higher than in a previous study [49], but the success rate was a little lower than those reported for sweet potato [43], rubber [44], and sesame [35]. The failure of 18 primer pairs to produce amplicons may have been due to the amplification with genomic DNA, the location of the primer across splice sites, large introns, chimeric primer, or poor-quality sequences [35]. The deviation of 45 amplicons from expected sizes may have been caused by the presence of introns, a lack of specificity, or assembly errors [44]. The 10 PCR products presented only one band, which might result from the primer design or the homozygosity of the loci in alfalfa germplasm.

Using 27 polymorphic EST-SSR markers, the *He* values ranged from 0.17 to 0.84, and most of the markers (88.9 %) had *He* values greater than 0.5. This result indicated a high level of polymorphism in alfalfa, as suggested previously [34,49]. This result may be due to a very high cross-pollination and autotetraploidy in this species. The 27 polymorphic EST-SSR markers are important for alfalfa research involving genetic diversity, relatedness, evolution, linkage mapping, cultivar protection, comparative genomics, and gene-based association studies. Next generation transcriptome sequencing provides plenty of sequences for molecular marker development. The 1,649 SSRs identified from our data will produce a wealth of markers for further genetic study. Based on these identified SSR-containing sequences, we will design more PCR primers and evaluate their polymorphism among alfalfa accessions with more individual plants in each accession, and provide a more valuable resource of genetic markers for future research in alfalfa. 

## Conclusions

This work presents a *de novo* transcriptome sequencing analysis of mixed RNAs from 15 different tissues. A total of 5.64 Gb of data were generated and assembled into 40,433 unigenes. Based on these sequences, EST-SSRs were predicted and analyzed. The 1,649 potential EST-SSRs predicted in this study provide a solid foundation for molecular marker development in alfalfa. A total of 27 polymorphic primer pairs successfully amplified fragments, revealing abundant polymorphisms between 10 alfalfa accessions. To our knowledge, this is the first study to use Illumina paired-end sequencing technology to investigate the global transcriptome of autotetraploid alfalfa and to assemble the reads without a reference genome. This valuable resource will provide a foundation for future genetic and genomic studies of autotetraploid alfalfa species.

## Supporting Information

Table S1 **Alfalfa germplasm accessions used in this study.**
(XLSX)

Table S2 **Sequences of 100 primer pairs for EST-SSR markers.**
(XLSX)

Table S3 **Annotation of 4,941 of 5,000 highly expressed sequences by significant BLASTn matches against the Nr database.**
(XLSX)

Table S4 **KEGG biochemical pathways for *M. sativa*.** This file contains the KEGG pathway and the number of unigenes involved in the KEGG pathway.
(XLSX)

Table S5 **Functional annotation of 1,494 alfalfa unigenes containing 1,649 potential di- to hexa-nucleotide SSRs with a minimum of 5 repeats for all motifs.**
(XLSX)

Table S6 **Nei's genetic identity (above diagonal) and genetic distance (below diagonal) between accessions.**
(XLSX)
